# Validity and reliability of the Spanish version of the DN4 (*Douleur Neuropathique 4 questions*) questionnaire for differential diagnosis of pain syndromes associated to a neuropathic or somatic component

**DOI:** 10.1186/1477-7525-5-66

**Published:** 2007-12-04

**Authors:** Concepcion Perez, Rafael Galvez, Silvia Huelbes, Joaquin Insausti, Didier Bouhassira, Silvia Diaz, Javier Rejas

**Affiliations:** 1 Unit of Pain, Hospital de la Princesa, Madrid, Spain; 2 Unit of Pain and Palliative Care, Hospital Universitario Virgen de las Nieves, Granada, Spain; 3 Neurological Research Unit, Hospital Nacional de Parapléjicos, Toledo, Spain; 4 Unit of Pain, Hospital Severo Ochoa, Leganés, Spain; 5 INSERM U-792, Hôpital Ambroise Paré, Boulogne-Billancourt, F-92100 France; 6 Université Versailles-Saint-Quentin, Versailles F-78035, France; 7 Department of Health Outcomes Research, Artac Bioestudios, Madrid, Spain; 8 Department of Health Outcomes Research, Medical Unit, Pfizer Spain, Alcobendas, Spain

## Abstract

**Background:**

This study assesses the validity and reliability of the Spanish version of DN4 questionnaire as a tool for differential diagnosis of pain syndromes associated to a neuropathic (NP) or somatic component (non-neuropathic pain, NNP).

**Methods:**

A study was conducted consisting of two phases: cultural adaptation into the Spanish language by means of conceptual equivalence, including forward and backward translations in duplicate and cognitive debriefing, and testing of psychometric properties in patients with NP (peripheral, central and mixed) and NNP. The analysis of psychometric properties included reliability (internal consistency, inter-rater agreement and test-retest reliability) and validity (ROC curve analysis, agreement with the reference diagnosis and determination of sensitivity, specificity, and positive and negative predictive values in different subsamples according to type of NP).

**Results:**

A sample of 164 subjects (99 women, 60.4%; age: 60.4 ± 16.0 years), 94 (57.3%) with NP (36 with peripheral, 32 with central, and 26 with mixed pain) and 70 with NNP was enrolled. The questionnaire was reliable [Cronbach's alpha coefficient: 0.71, inter-rater agreement coefficient: 0.80 (0.71–0.89), and test-retest intra-class correlation coefficient: 0.95 (0.92–0.97)] and valid for a cut-off value ≥ 4 points, which was the best value to discriminate between NP and NNP subjects.

**Discussion:**

This study, representing the first validation of the DN4 questionnaire into another language different than the original, not only supported its high discriminatory value for identification of neuropathic pain, but also provided supplemental psychometric validation (i.e. test-retest reliability, influence of educational level and pain intensity) and showed its validity in mixed pain syndromes.

## Background

Appropriate therapeutic management of pain requires an accurate diagnosis, distinguishing its cause and origin. According to the International Association for the Study of Pain (IASP), neuropathic pain (NP) is defined as a pain initiated or caused by a primary lesion (or dysfunction) of the nervous system, and comprises a very large group of neurological conditions including diabetic and other sensory polyneuropathies, trigeminal neuralgia, post-herpetic neuralgia, stroke, spinal cord injury, and multiple sclerosis, as well as common conditions such as lumbar or cervical radiculopathies, traumatic or postsurgical nerve injuries, etc [1-4]. Virtually any condition damaging the nervous tissues or causing neuronal dysfunction may cause NP [5], suggesting that NP prevalence is high in the general population. Recent data suggest that NP might affect up to 5%–8% of the general population [6-10].

Due to the lack of validated or consensual diagnostic criteria, diagnosis of NP has traditionally been based on identification of the neurological lesion through the medical history, neurological examination, and appropriate electrophysiological or imaging investigations [11,12]. A series of recent studies [13-18] confirmed that chronic pain associated to a nerve lesion has specific clinical characteristics and showed that the combinations of selected symptoms and signs have a very high discriminant value for identification of this category of pain. This was the basis for the development and validation of screening tools in the form of simple questionnaires that could be helpful both in daily practice and clinical research. Interestingly, although they were developed in parallel in different countries and languages (English, German, French), most items (i.e. pain descriptors) included in these clinical tools are similar [19]. Thus, despite the specificities associated to the description of chronic pain in different cultures, the symptom-based approach for diagnosis of neuropathic pain appears to have transcultural validity.

In order to test this hypothesis, this study analyzed the psychometric properties of the DN4 questionnaire translated into Spanish. The DN4 questionnaire was originally developed and validated in French [15]. It is a clinician-administered questionnaire consisting of 10 items. Seven items related to pain quality (i.e. sensory and pain descriptors) are based on an interview with the patient, and 3 items based on the clinical examination are related to the presence or absence of touch or pinprick hypoesthesia and tactile allodynia. The DN4 questionnaire has very good sensitivity (83%) and specificity (90%) for identification of chronic pain associated to a lesion in the nervous system (either peripheral or central).

Thus, the aim of this study was not only the translation and cultural adaptation of the DN4 questionnaire into Spanish, but to provide also a supplemental psychometric validation of the questionnaire. In particular, in addition to the general diagnostic properties of our translated questionnaire (i.e. sensitivity and specificity), its test-retest reliability was verified, and the influence of various factors, such as pain intensity and educational level, on the DN4 results was analyzed. The DN4 was also administered to a group of patients with a combination of neuropathic and non-neuropathic pain to support its validity in mixed pain syndromes.

## Methods

The DN4 questionnaire [15] consists of a total of 10 items grouped in 4 sections [Additional file 1]. The first seven items are related to the quality of pain (burning, painful cold, electric shocks) and its association to abnormal sensations (tingling, pins and needles, numbness, itching). The other 3 items are related to neurological examination in the painful area (touch hypoesthesia, pinprick hypoesthesia, tactile allodynia). A score of 1 is given to each positive item and a score of 0 to each negative item. The total score is calculated as the sum of all 10 items, and the cut-off value for the diagnosis of neuropathic pain is a total score of 4/10. All questions are related to pain which is the claim for current medical consultation.

### Translation of the questionnaire

The adaptation into Spanish of the DN4 questionnaire and the subsequent assessment of its psychometric properties (i.e. reliability and validity) have been performed following the traditional recommendations for adaptation and validity of health questionnaires and diagnostic tests [20-25]. In the preparation phase, an expert panel was selected, the original questionnaire was translated, the sample was defined, and the study documentation was prepared. The adaptation procedure was monitored by a five-expert panel including three specialists in pain management, an expert in methodology, and an expert in clinical research. These experts reviewed the translation and monitored the adaptation process. As the original version of the questionnaire was well defined and structured, the expert panel did not consider it necessary to redefine its sections or reformulate the original questions. No cultural bias that could be equivocal or non-translatable was detected in the original instrument. Questionnaire translation was commissioned to two independent professional translators (philologists, Spanish natives) to have two parallel translations. The expert panel pooled both translations into a single version that was tested in a sample of 12 patients [other than those included in the validation sample; 7 (57%) males, mean age 64.5 ± 15.3 years (± standard deviation)] to assess initial feasibility and potential understanding problems. The final version was back-translated into French by two other professional translators (different from the first two translators, and French natives) and submitted to the original author (Dr. D Bouhassira), who proposed a change in the translation of one of the descriptors in the pain scale to certify agreement with the original instrument.

### Patients

Patients of both sexes with chronic pain for more than 3 months aged 18 years or over, with an adequate cultural level to understand health questionnaires administered in Spanish were included in this study. Chronic pain could be of a neuropathic, non-neuropathic, or mixed (i.e. both neuropathic and non-neuropathic components) origin. Pain was classified based on the medical history, physical examination, and any procedures (laboratory tests, electrophysiology, imaging, etc.) considered appropriate by the clinicians to establish the diagnosis of the type of pain. All patients gave their written informed consent before entering the study. Tables 1 and 2 show the main sociodemographic data and clinical conditions responsible for pain in the study sample.

**Table 1:** Demographic characteristics of study patients, overall and by the main subgroups with (NP) and without (NNP) associated neuropathic component.

**Variable**	**Total**	**NP**	**NNP**
N	158	99 (62.7%)	59 (37.3%)
Sex, females; n (%)	93 (58.9%)	46 (47%)^†^	47 (80%)
Race (Caucasian); n (%)	149 (95.5%)	93 (94%)	56 (98%)
Age (years)	60.1 (15.9)	57.2 (15.2)^†^	64.9 (16.1)
BMI (kg/m^2^)	27.3 (4.6)	27.3 (4.1)	27.2 (5.4)
Drug therapy;			
n (%):	145 (91.8%)	89 (90%)	56 (95%)
no. of drugs: ^a^	2.0 (1.0 – 3.0)	2.0 (1.0 – 3.0)	2.0 (1.0 – 3.0)
Educational level; n (%)^†^			
No studies:	39 (25.0%)	19 (19%)	20 (35%)
Primary school:	69 (44.2%)	45 (46%)	24 (41%)
High school:	14 (9.0%)	12 (12%)	2 (3%)
Vocational training:	16 (10.3%)	14 (14%)	2 (3%)
Graduate studies:	18 (11.5%)	8 (8%)	10 (17%)

SF-MPQ scoring;			
Sensory dimension (0 – 33):	12.4 (6.4)	13.1 (6.0)	11.3 (6.8)
Affective dimension (0 – 12):	4.9 (3.6)	4.8 (3.6)	5.2 (3.6)
Total score (0 – 45):	17.4 (8.3)	17.9 (8.1)	16.5 (8.8)
Pain in the previous week (VAS; 0 – 100):	65.4 (20.7)	65.6 (22.2)	65.0 (18.2)

Values are given as mean (standard deviation) or frequency (%) based on the number of valuable cases (information available). BMI = Body Mass Index, SF-MPQ = Short Form McGill Pain Questionnaire. ^a^Median (1st – 3rd quartile), ^† ^p < 0.05 vs. NNP group.

**Table 2:** Distribution of the most common etiological causes of neuropathic pain (peripheral and central), mixed pain and non-neuropathic pain in the study patients.

**Neuropathic Pain **(n = 57)		**Mixed Pain **(n = 42)		**Non Neuropathic Pain **(n = 59)	
Peripheral	25 (44%)	Radiculopathys	26 (62%)	Osteoarthritis	50 (85%)
Polyneuropathy	6 (24%)	Entrapment syndrome	5 (12%)	Spondylolisthesis	4 (7%)
Neuralgia*	16 (64%)	Atypical facial pain	3 (7%)	Mechanical low back pain	5 (8%)
Phantom member syndrome	3 (12%)	Others**	8 (19%)		
Central	32 (56%)				
Spinal cord injury	26 (81%)				
Post-stroke	4 (13%)				
Multiple sclerosis	2 (6%)				

Includes trigeminal (n = 5) and post-herpetic (n = 11) neuralgia. ** Includes complex regional pain syndrome, parestethic meralgia and cancer.

### Study design

In addition to the DN4 questionnaire, average daily pain intensity was measured using a 100 mm visual analogue scale (VAS), and the short-form McGill Pain Questionnaire (SF-MPQ) [26] was used to assess the sensory and affective components of pain. The SF-MPQ includes 15 items related to the presence and severity (assessed with a 4-point Likert scale) of pain descriptors associated to the affective (4 items) or sensory (11 items) dimensions of chronic pain.

All investigators were pain experts. At each participating center, the main investigator collected the sociodemographic and clinical data required for patient characterization and was required to classify patients into two broad diagnostic categories corresponding to NP and NNP, and to further subcategorize NP into peripheral, central or mixed pain. The diagnosis of the principal investigator was considered as the reference (gold standard) diagnosis. Patients were then separately seen within 2 days by two other investigators (raters A and B) blinded to the diagnosis proposed by the principal investigator, who administered the DN4 and SF-McGill questionnaires.

This study was conducted according with usual standard of care in each participant centre, and it did not involve any drug or therapeutic management. Nevertheless, the study design was approved by the IRB of the Universidad Autónoma de Madrid and was conducted in compliance with the Helsinki Declaration for research in humans.

### Psychometric measurements and statistical analysis

#### Reliability

The internal consistency of the Spanish version of the DN4 questionnaire was separately established for raters A and B by calculating Cronbach's α coefficient that assesses the contribution of each item to the precision of measurements by the instrument. Cronbach's α coefficient was assessed in the complete questionnaire and after removing each item from it to assess the independent contribution of each item to the measurement error in the instrument.

Inter-rater reliability was assessed by the agreement of the results obtained by raters A and B for each item and the total score of the DN4 questionnaire. Agreement was determined by calculating the Cohen's kappa coefficient. Inter-rater agreement was also determined by calculating the intra-class correlation coefficient of the total scores assigned by each pair of raters to the same subject, and the answers to each individual item, using a two-factor model and mixed effects.

In a subject sub-sample (n = 68), the test-retest reliability of the questionnaire was assessed by a third administration, after at least 48 hours, of the DN4 scale by rater B. Stability of the questionnaire was analyzed by measuring the intra-class correlation coefficient between the scores of clinicians completing test and retest and using Cohen's kappa coefficient of agreement for the total retest sample and in subgroups by type of pain.

#### Validity

A ROC (receiver operating characteristic) curve analysis was performed to determine the cut-off value of the questionnaire score providing the best values of sensitivity and specificity for NP diagnosis and determination of Youden's index for the total patient sample included in the study. This was repeated in two sub-samples excluding either patients with mixed pain only or patients with mixed and subjects with central NP (just to test the instrument in patients with peripheral NP only). For each cut-off value of the scale score, in addition to sensitivity and specificity and Youden's index, the positive (PPV) and negative predictive value (NPV), the kappa coefficient of agreement with the diagnosis according to the standard criterion or reference diagnosis, and the area under the curve (AUC) calculated by the trapezoid method and its significance level were estimated. The 95% confidence intervals (95% CI) of the estimators of the cut-off point selected as optimum were calculated. Validity indicators (sensitivity, specificity, PPV, and NPV) were recalculated individually for the presence of each descriptor symptom of the DN4 questionnaire. The Youden's index was calculated by the equation: sensitivity + specificity - 1 [27].

Descriptive statistics were prepared for the variables tested in this study to assess central positition, dispersion, and distribution of variables tested by the Kolmogorov- Smirnov test. A Student's t test for independent groups or a Mann-Whitney's U test was used in the case of a non-normal distribution to compare continuous or ordinal variables respectively between patients with NP and NNP. All statistical tests were two-tailed, and an α error of < 0.05 was accepted as statistically significant. Data were analyzed using SPSS version 12.0 statistical software.

## Results

### Sample description

The study was conducted from June to November 2005. Table 1 summarizes the clinical and sociodemographic characteristics of the 158 patients, 99 with NP and 59 with NNP, included in the study, and Table 2 details pain etiology. NP was peripheral in 25 (25%) patients, central in 32 (32%), and mixed in 42 patients (42%). The proportion of women and the mean age were significantly higher in the NNP group, but the two groups were homogenous in terms of educational level, pain intensity and analgesic treatment (Table 1). The SF-MPQ total score and subscores by dimension were not statistically significant between patients with or without NP (Table 1).

### Analysis of psychometric properties of the Spanish version of the DN4 questionnaire

#### Internal consistency, inter-rater agreement and test-retest reliability

Table 3 summarizes the values of the indicators related to reliability of the Spanish version of the DN4 questionnaire. Our data support the reliability of the instrument both in terms of internal consistency (Cronbach's α of approximately 0.7 in both observers) and inter-rater reliability (Cohen's kappa coefficients ranging from 0.68 and 0.79) or questionnaire stability (intra-class correlation coefficients ranging from 0.92 and 0.95). Cronbach's α coefficients did not improve when each scale item was successively removed (values ranging from 0.60 and 0.71), which justifies the contribution of each item to measure the concept measured by the questionnaire (Table 3).

Inter-rater reliability, which is essential for an instrument that contains a semi-structured interview subject to interviewer interpretation, was good to very good. The total scores in each diagnostic group did not show statistically significant differences, and the intra-class correlation coefficient values ranged from 0.84 and 0.93 (Table 3). Calculation of the kappa coefficients supported the inter-rater agreement regarding diagnostic classification. In addition, retest results confirmed that the DN4 questionnaire has good test-retest stability, with intra-class correlation coefficients ranging from 0.92 and 0.95.

**Table 3:** Internal consistency, inter-rater agreement, and test-retest reliability of the Spanish version of the DN4 questionnaire.

	**Inter-rater agreement**			
	
	**Rater A**	**Rater B**	**ICC **(95% CI)	**Kappa**^§ ^(95% CI)
	
Total sample (n = 158)	4.1 (2.3)^†^	4.2 (2.5)	0.926 (0.899–0.946) ^‡^	0.79 (0.69–0.89)
NP (n = 99)	5.0 (2.2) ^†^	5.1 (2.4)	0.925 (0.888–0.949) ^‡^	0.68 (0.49–0.86)
NNP (n = 59)	2.6 (1.8)^†^	2.7 (2.0)	0.840 (0.730–0.905) ^‡^	0.72 (0.50–0.93)
			
	**Internal consistency **(Cronbach's α^1^)			
			
	0.65	0.71		
	
	**Test-retest reliability**			
	
	**Test**	**Re-test**	**ICC **(95% CI)	**Kappa**^§ ^(95% CI)
	
Retest sample (n = 67)	3.7 (2.5)^†^	3.6 (2.6)	0.949 (0.916–0.969) ^‡^	0.79 (0.64–0.94)
NP (n = 37)	4.7 (2.4) ^†^	4.4 (2.5)	0.952 (0.904–0.976) ^‡^	0.75 (0.53–0.98)
NNP (n = 30)	2.5 (2.0)^†^	2.7 (2.4)	0.923 (0.840–0.963) ^‡^	0.78 (0.56–1.00)

NP = Neuropathic Pain, NNP = Non-Neuropathic Pain (somatic), ICC = Intra-class Correlation Coefficient, 95% CI = 95% confidence interval, ^1^Coefficients for the entire questionnaire (10 items) ^†^p > 0.05 between raters (Friedman's paired test), ^‡ ^p < 0.001 (p value for intra-class correlation coefficient), ^§^Kappa using a cut-off value > 4 pts.

#### Validity

Table 4 gives the results of scale validity as a diagnostic method for NP, comparing its properties first in the total sample of NP patients, and then after successively removing patients with mixed pain and with central NP. In all cases, ROC curve analysis identified a score of 4 as the best cut-off value discriminating between NP and NNP (values of the area under the curve ranging from 0.85 to 0.87, p < 0.001 in all cases, and the highest values of the Youden's index). This cut-off point showed, in all cases compared, values of 70% or higher (except for PPV in the case of peripheral NP) in indicators of sensitivity, specificity, positive predictive value (PPV), and negative predictive value (NPP), which anticipates a low percentage of both false positive and false negative results when the questionnaire is used.

**Figure 1:**
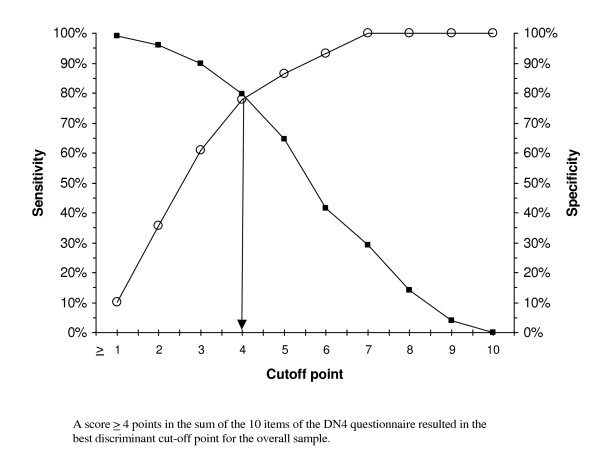
Cut-off point in the DN4 questionnaire optimizing the sensitivity and specificity values for discriminating between neuropathic pain and non-neuropathic pain in the overall sample.

**Figure 2:**
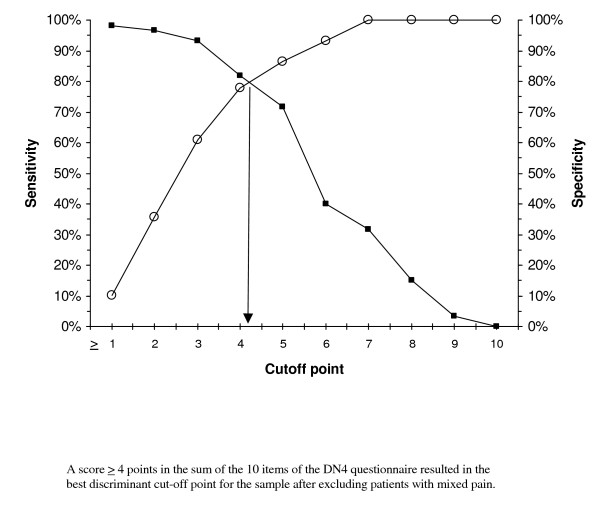
Cut-off point in the DN4 questionnaire optimizing the sensitivity and specificity values for discriminating between pure neuropathic pain (excluding patients with mixed pain) and non-neuropathic pain.

**Figure 3:**
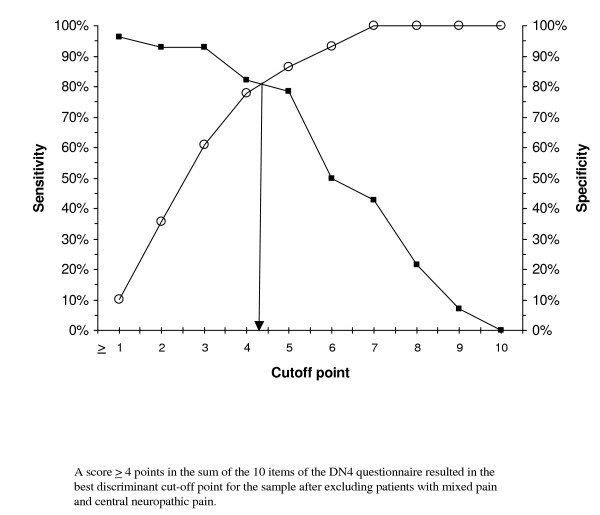
Cut-off point in the DN4 questionnaire optimizing the sensitivity and specificity values for discriminating between peripheral neuropathic pain (excluding patients with mixed pain and central neuropathic pain) and non-neuropathic pain.

The graphs plotting the cut-off point optimizing the sensitivity and specificity values consistently show a cut-off point ≥ 4 points as the most appropriate value for discriminating between NP and NNP in both the total sample and after removing patients with mixed pain and central NP (Figures 1, 2 and 3 respectively).

The indicators of the validity of the DN4 questionnaire to differentiate NP from NNP were good in all cases, regardless of whether the analysis included all patients with NP and NNP or patients with mixed pain or central NP were removed. The improvement seen in sensitivity values when patients who could introduce classification errors were removed was moderate, though both positive and negative predictive values showed more marked changes, improving as expected as patients who could potentially add errors (case of negative predictive value) were removed, and worsening when such patients were included (case of the positive predictive value, Table 4).

**Table 4:** Comparison of the psychometric properties of validity for differential diagnosis of neuropathic pain vs non-neuropathic pain for a cut-off value ≥ 4 points in the Spanish version of the DN4 questionnaire, in the overall sample and in patients with pure neuropathic pain (excluding those with mixed pain) and with peripheral neuropathic pain (excluding patients with mixed and central pain).

	**All patients **(n = 158)	**Mixed pain excluded **(n = 116)	**Mixed and central pain excluded **(n = 84)
**Youden index**	0.58	0.60	0.60
**Sensitivity (%)**	79.8% (71%–87%)	81.7% (70%–91%)	82.1% (63%–94%)
**Specificity (%)**	78.0% (65%–88%)	78.0% (65%–88%)	78.0% (65%–88%)
**PPV (%)**	85.9% (77%–92%)	79.0% (67%–88%)	63.9% (46%–79%)
**NPV (%)**	69.7% (57%–80%)	80.7% (68%–90%)	90.2% (79%–97%)
**AUC**	0.85 (0.79–0.91) ^†^	0.87 (0.80–0.93) ^†^	0.87 (0.78–0.96) ^†^
			
**% of agreement**	79.1% (72%–85%) ^§^	79.8% (72%–87%) ^§^	79.3% (69%–87%) ^§^
**Kappa's Agreement**	0.56 (0.43–0.70) ^‡^	0.60 (0.45–0.75) ^‡^	0.56 (0.39–0.74) ^‡^

Youden index = Sensitivity + Specificity – 1, 95% confidence interval in brackets, PPV = Positive Predictive Value, NPV = Negative Predictive Value, AUC = Area under the curve (ROC curve analysis). ^†^p < 0.001 (p value in ROC curve analysis), ^§^p > 0.05 between reference diagnosis and DN4 classification for a cut off value ≥ 4 pts (McNemar's test), ^‡ ^p < 0.001 (p value for Cohen's kappa of agreement between reference diagnosis and DN4 classification for a cut off value ≥ 4 pts).

#### Validity in subgroups according to pain severity and educational level

Table 5 shows the validity properties of the DN4 questionnaire in patient subgroups depending on mean pain severity in the week prior to questionnaire administration and depending on the educational level of patients. Questionnaire performance was not significantly influenced by the educational level, but was significantly decreased in patients with mild pain.

**Table 5:** Properties of validity for diagnosis of pain with neuropathic involvement in different subgroups by pain severity and educational level in the overall sample.

**Cut-off value ≥ 4 points **	**Sensitivity (%)**	**Specificity (%)**	**PPV (%)**	**NPV (%)**	**Kappa**
**Pain severity (VAS)**					
Mild; < 40 mm (n = 12)	55.6	66.7	83.3	33.3	0.17^‡^
Moderate; ≥ 40 and < 70 mm (n = 65)	85.0	84.0	89.5	77.8	0.68^†††^
Severe; ≥ 70 mm (n = 81)	80.0	74.2	83.3	69.7	0.54^†††^

**Educational level**					
No studies (n = 39)	81.3	73.9	68.4	85.0	0.54^††^
Primary school (n = 69)	77.1	81.0	90.2	60.7	0.53^†††^
High school (n = 14)	83.3	100	100	50.0	0.59^†^
Vocational training (n = 16)	78.6	100.0	100	40	0.48^†^
Graduate studies (n = 18)	87.5	70.0	70.0	87.5	0.56^†^

VAS = Visual Analogue Scale for pain (0 – 100 mm) of McGill short pain questionnaire, PPV = Positive Predictive Value, NPV = Negative Predictive Value. ^‡ ^p > 0.05; ^†^p < 0.05; ^††^p < 0.01; ^†††^p < 0.001. Two patients did not report their educational level.

## Discussion

This study represents the first validation of the DN4 questionnaire, originally validated into French, into another language. Overall, the high discriminant value of the Spanish version of the DN4 questionnaire for identification of neuropathic pain was confirmed. In addition, our study provided new information about the test-retest reliability of the questionnaire, the influence of educational level and pain intensity on its performance, and its validity in mixed pain syndromes.

Identification of neuropathic components is crucial for characterization of chronic pain patients and to adapt the therapeutic approach, given the differential response of neuropathic pain to analgesic treatment [7,28,29]. Therefore, a better identification of neuropathic pain should improve the therapeutic outcome. Several diagnostic and screening tools have been developed and validated over the past few years for this purpose [19]. All these screening/diagnostic tools make use of similar descriptors to discriminate neuropathic patients from other chronic pain patients with up to 80%–90% sensitivity and specificity. The DN4, initially developed and validated in 160 French-speaking patients, has very high diagnostic properties [15] and is one of the easiest tools to use. In particular, its scoring is very simple because, unlike in other tools, the items are not "weighted". In the original study, only patients with "pure" neuropathic pain (i.e. not "mixed pain" syndromes) of at least moderate intensity (i.e. ≥ 4 out of 10) were included, and the test-retest reliability was not investigated. Thus, in addition to the linguistic validation of the DN4 questionnaire into Spanish, it appeared of interest to provide additional psychometric validation of this questionnaire.

The reliability and validity of the Spanish version of the DN4 questionnaire were confirmed; in particular, our analyses showed that, similarly to the French version, the best cut-off value corresponds to a total score of 4 out of 10. The overall performance of the Spanish DN4 questionnaire was very good, although specificity and sensitivity appeared to be slightly lower as compared to the original version. These differences might reflect linguistic specificities and cultural differences, but they may also result from methodological disparities (e.g. differences in the clinical characteristics of patients) between both studies. In particular, our analysis of the properties of the questionnaire according to pain intensity revealed significantly lower sensitivity and specificity in patients with mild pain intensity. The influence of pain intensity on the psychometric properties of neuropathic pain screening tools had not been previously tested with the DN4 or with other questionnaires. The data reported here suggest that these tools might be much less discriminant and should therefore be used with caution in patients with reduced pain intensity. However, this would have to be confirmed in further studies with the DN4 or other diagnostic questionnaires, because the subgroup of patients with mild pain intensity was relatively small in this study. By contrast, our results suggest that the discriminant value of the DN4 questionnaire did not depend on the educational level, confirming that these simple symptom-based questionnaires are easily understood and that their administration should not be restricted to highly educated patients. A supplemental validation investigated in this study concerned the stability over time (i.e. test-retest reliability) of the DN4 questionnaire, which was excellent in our patients with or without NP.

The results of the DN4 were similar in patients with "pure" neuropathic pain or mixed pain syndromes, (i.e. the combination of neuropathic and non neuropathic pain in the same patient). Thus, the present data suggest that the neuropathic component of the "mixed pain" syndrome has clinical characteristics similar to those of "pure" neuropathic pain. Interestingly, this is consistent with the results recently reported by Freynhagen et al [17], showing that radiculopathies associated with mixed pain syndromes have clinical characteristics similar to those of definite neuropathic pain syndromes. In addition, the statistical estimators of validity of the test remained mostly unchanged after removing both the subgroups of patients with mixed pain and with central pain, leaving pure peripheral neuropathic pain only. This suggests that the error level of the questionnaire will remain largely unchanged. More generally, our data tend to support the interest of NP screening tools in the clinical setting. These tools should be of particular interest for both Primary Care Physicians (PCPs) and other non pain specialists to identify NP, diagnose its cause, and adapt the analgesic treatment. Thus, screening tools like DN4 should help PCPs to take a more pro-active role in appropriate treatment selection, thus avoiding early therapeutic failure and patient suffering.

## Conclusion

The results of this study support the transcultural validity of the DN4 questionnaire. More generally, our data tend to confirm the interest of symptom-based diagnostic tools for identification of the neuropathic pain component. These simple clinical tools may be used not only in daily practice, but also in the clinical research setting (e.g. epidemiological studies). Future studies directly comparing the performance of the different available tools are warranted.

## Abbreviations

DN4: Douleur Neuropathique 4 questions;

NP: Neuropathic pain;

NNP: Non Neuropathic pain;

IASP: International Association for Study of Pain;

VAS: Visual Analogue Scale;

SF-MPQ: Short-form McGill Pain Questionnaire;

ROC: Receiver Operating Characteristic;

PPV: Positive Predictive Value;

NPV: Negative Predictive Value;

AUC: Area under the curve;

PCP: Primary Care Physicians.

## Competing interests

Javier Rejas is employed at Pfizer Spain. The author(s) declare that they have no competing interests.

## Authors' contributions

CP and JR designed the study and were responsible for the idea of the study. CP, RG, SH and JI participated in patient enrrollment and manuscript elaboration. DB wrotte the manuscript in part and was responsible for consultation and scientific support of the project. SD was responsible for monitoring, logistic and evaluation of data. All authors read and approved the final manuscript.

## Supplementary Material

Additional file 1 Cuestionario DN4. This appendix includes the Spanish version of DN4 questionnaire. Click here for file
