# Supplementary material for: Validity and reliability of the Spanish version of the DN4 (Douleur Neuropathique 4 questions) questionnaire for differential diagnosis of pain syndromes associated to a neuropathic or somatic component
Source: Health Qual Life Outcomes. 2007 Dec 4;5:66. doi: 10.1186/1477-7525-5-66 (PMC2217518; doi:10.1186/1477-7525-5-66)
Supplement: Additional file 1 — Cuestionario DN4. This appendix includes the Spanish version of DN4 questionnaire. [file 1477-7525-5-66-S1.doc]

**Cuestionario DN4**

(Versión española del cuestionario Dolour Neuropathique 4*[[1]](#footnote-2)*)

Responda a las cuatro preguntas siguientes marcando sí o no en la casilla correspondiente.

**ENTREVISTA AL PACIENTE**

Pregunta 1: ¿Tiene su dolor alguna de estas características?

|  |  | Si |  | No |
| --- | --- | --- | --- | --- |
| 1 | Quemazón |  |  |  |
| 2 | Sensación de frío doloroso |  |  |  |
| 3 | Descargas eléctricas |  |  |  |

Pregunta 2: ¿Tiene en la zona donde le duele alguno de estos síntomas?

|  |  | Si |  | No |
| --- | --- | --- | --- | --- |
| 4 | Hormigueo |  |  |  |
| 5 | Pinchazos |  |  |  |
| 6 | Entumecimiento |  |  |  |
| 7 | Escozor |  |  |  |

**EXPLORACIÓN DEL PACIENTE**

Pregunta 3: ¿Se evidencia en la exploración alguno de estos signos en la zona dolorosa?

|  |  | Si |  | No |
| --- | --- | --- | --- | --- |
| 8 | Hipoestesia al tacto |  |  |  |
| 9 | Hipoestesia al pinchazo |  |  |  |

Pregunta 4: ¿El dolor se provoca o intensifica por?

|  |  | Si |  | No |
| --- | --- | --- | --- | --- |
| 10 | El roce |  |  |  |

1. *Versión Francesa (Francia): Bouhassira D, et al. Pain 2005; 114: 29-36.*

   *Versión Española (España): Pérez C, et al. EFIC 2006.*  [↑](#footnote-ref-2)
